# Age-Related Patterns of Symptoms and Risk Factors in Acute Coronary Syndrome (ACS): A Study Based on Cardiology Patients’ Records at Rehman Medical Institute, Peshawar

**DOI:** 10.7759/cureus.58426

**Published:** 2024-04-16

**Authors:** Nouman Anthony, Amir Hassan, Usman Ghani, Omar Rahim, Moula Ghulam, Neha James, Zarbakhta Ashfaq, Saad Ali, Amna Siddiqui

**Affiliations:** 1 General Medicine, Rehman Medical Institue, Peshawar, PAK; 2 Medicine, Shaukat Khanum Memorial Cancer Hospital and Research Centre, Peshawar, PAK; 3 Cardiology, Northwest General, Peshawar, PAK; 4 Internal Medicine, Naseer Teaching Hospital, Peshawar, PAK; 5 Medicine, Rehman Medical Institute, Peshawar, PAK; 6 General Medicine, Rehman Medical Institute, Peshawar, PAK; 7 Medicine, Rehman Medical Institute, Peshawar , PAK

**Keywords:** cardio vascular disease, risk factors of acs in pakistan, gender and acs, age and acs, acs ( acute coronary syndrome )

## Abstract

Introduction

Acute Coronary Syndrome (ACS) is a critical condition characterized by reduced blood flow to the heart and includes various conditions such as ST-elevation myocardial infarction, non-ST elevation myocardial infarction, and unstable angina.

Objectives

The aim of this study was to investigate age-related patterns of symptoms and risk factors in ACS patients and to evaluate how diagnostic test results differ among various age groups of ACS patients.

Methodology

This retrospective study was conducted from May to November of 2023 on patients with acute coronary syndrome admitted to the cardiology ward of Rehman Medical Institute (RMI), Peshawar. The sample size was 137 ACS-diagnosed patients based on the inclusion and exclusion criteria. After getting ethical approval from the institutional ethical approval board, data were collected for the entire year of 2022 based on proforma with the variables demographic data, troponin I level, presented symptoms, and associated co-morbidities of the patients. The inclusion criteria were patients of all genders, patients diagnosed with Acute Coronary Syndrome (ACS), and patients whose records were available in the cardiology department of Rehman Medical Institute.

Results

The results show that ACS is more prevalent in the age group of 50-69 years (p=0.037) and is significantly more common in males (p=0.019). Chest pain emerged as the predominant symptom, with a significant association of p=0.029 between chest pain and patients of ACS in the age group 30-49 years. While raised troponin I levels were prevalent across all age groups. Moreover, specific risk factors such as diabetes mellitus, hypertension, and family history of CAD showed the significance of p= 0.04, p=0.006, and p=0.021, respectively, with the age group 50-69 years old.

Conclusion

This study highlights the importance of considering age and gender in ACS management and provides insights into age-related patterns of symptoms and risk factors, which can contribute to optimizing preventive strategies and improving patient care. Further research is needed to explore the underlying mechanisms and assess long-term outcomes in different age groups.

## Introduction

Acute Coronary Syndrome (ACS) encompasses various conditions that result in reduced blood flow to the heart, including ST-elevation myocardial infarction, non-ST elevation myocardial infarction, and unstable angina [1]. ACS is a critical condition that necessitates immediate attention. Doctors diagnose ACS by considering the patient's medical history, paying close attention to chest pain, and using various techniques, such as biochemical, electrocardiographic, and imaging methods [2]. The primary symptom of ACS is chest pain, a heavy pressure or squeezing sensation accompanied by a burning feeling and difficulty breathing. This pain may spread to the left shoulder, neck, or arm and tends to worsen over time, often triggered by exercise or stress. Notably, it can also occur without an apparent cause [3].

Every year, approximately 7 million people worldwide, including 1 million hospitalized patients in the US, are diagnosed with ACS, with a higher prevalence among older individuals [4,5]. Compared to Western populations, the Asian region has been found to bear a more significant burden of cardiovascular disease (CVD), primarily impacting economically disadvantaged populations concentrated in South Asia [6]. Previous studies have investigated the impact of age on various forms of ACS [7]. Notably, statistics from the American Heart Association (AHA) reveal that 83% of coronary artery disease (CAD)-related deaths occur in patients aged 65 or older [8]. ACS mortality rates increase by an odds ratio of 1.7 for every 10-year increment beyond the age of 65. Elderly patients possess more cardiovascular risk factors and a higher burden of ischemic disease compared to younger patients [9]. Low-middle-income countries (LMICs) bear a significant burden, and Pakistan is particularly impacted, with a prevalence of 15.31% in the country. Among various health concerns in Pakistan, ischemic heart disease (IHD) stands out as the primary cause of death [10]. 

Our study aims to examine the age-related patterns of symptoms and risk factors in patients with ACS. While variations in ACS presentation and diagnostic challenges exist across different age groups, the relationship between age and the occurrence of atypical and typical ACS symptoms has received limited attention. Understanding these variations is crucial for accurate diagnosis and effective management of ACS in patients of different ages. Also, to evaluate how diagnostic test results differ among various age groups of ACS patients. Moreover, identifying age-specific risk factors associated with ACS is essential for developing tailored prevention and management strategies. By considering patients' unique characteristics and needs in different age groups, we can optimize patient care and enhance ACS outcomes.

## Materials and methods

Methodology

This retrospective, cross-sectional study aimed to investigate age-related patterns of symptoms and risk factors in patients diagnosed with Acute Coronary Syndrome (ACS). The study utilized data collected from the cardiology patient records at Rehman Medical Institute, Peshawar, Pakistan. The study duration was from May to November of 2023. The sample size was 137 ACS-diagnosed patients based on inclusion and exclusion criteria. The inclusion criteria were patients of all genders, patients diagnosed with Acute Coronary Syndrome (ACS), and patients whose records were available in the cardiology department of Rehman Medical Institute. The exclusion criteria included patients with incomplete or missing records, patients with a history of other cardiovascular diseases (dysrhythmias, exacerbated heart failure, valvular heart diseases), and patients with incomplete questionnaire responses. The data were collected from the cardiology patient records of Rehman Medical Institute for the entire year of 2022. A questionnaire was developed to collect relevant information from the patient records which included demographic details, medical history, symptoms, risk factors, and relevant diagnostic test results. Trained personnel extracted the required information from the patient records and completed the questionnaire accordingly. Data entry was conducted using a secure and reliable database system. The study adhered to ethical guidelines and principles of data protection. Patient data was anonymized and kept confidential. The study protocol was submitted for ethical review and approval by the institutional review board.

Statistical analysis

The collected data were entered into the SPSS software version 22.0 for statistical analysis. Descriptive statistics were used to summarize the study population's demographic characteristics, symptoms, and risk factors. The chi-square test, or Fisher's exact test, was used to examine the association between age groups and symptoms/risk factors. A logistic regression analysis may have been performed to identify independent predictors of ACS in different age groups. Statistical significance was set at p < 0.05. The variables of Interest were age: categorical variable (divided into predefined age groups, e.g., 30-49, 50-69, 70+), symptoms: Presence or absence of specific symptoms (e.g., chest pain, shortness of breath, fatigue, etc.), risk factors: Presence or absence of established risk factors (e.g., smoking, hypertension, diabetes, hyperlipidemia, family history, etc.), diagnostic test results: ECG findings, troponin levels, other relevant variables: include gender, medical history. After analysis, the data were presented in the form of tables. 

## Results

This retrospective, cross-sectional study aimed to investigate the impact of age on ACS and its associated risk factors. The study included 137 patients diagnosed with acute myocardial infarction (AMI) and examined their demographic characteristics. The majority of patients (58.3%) fell into the 50-69 years age range, followed by 70-89 years (21.2%) and 30-49 years (19%). The lowest number of patients was in the 10-29 years age group (0.7%). Gender distribution revealed that 60% of the patients were male and 40% were female, with the highest proportion of males in the 50-69 age range (55%) and females in the 50-69 years age range (63.6%). Significant associations were found between age (p = 0.037) and gender (p = 0.019) with ACS, as seen in Table 1.

**Table 1:** Demographic data and symptoms SOB = shortness of breath; N/V = nausea and vomiting; GBW = generalized body weakness.

Variable	Overall	10-29 age group	30-49 age group	50-69 age group	70-89 age group	90-99 age group	P-value
Age (n,%)	137 (100%)	1 (0.7%)	26 (19%)	80 (58.3%)	29 (21.2%)	1 (0.7%)	0.037
Gender (n,%)							
Male	82 (60%)	0 (0%)	19 (73%)	45 (56.2%)	17 (58.6%)	1 (100%)	0.019
Female	55 (40%)	1 (100%)	7 (26.9%)	35 (43.7%)	12 (41.3%)	0 (0%)	
Symptoms (n,%)							
Chest Pain	96 (70%)	1 (100%)	23 (88.4%)	57 (71.2%)	15 (51.7%)	0 (0%)	0.029
SOB	51 (37.2%)	1 (100%)	8 (30.7%)	29 (36.2%)	13 (44.8%)	0 (0%)	0.031
Palpitations	4 (2.9%)	0 (0%)	0 (0%)	3 (3.7%)	1 (3.4%)	0 (0%)	0.938
Syncope	2 (1.4%)	0 (0%)	0 (0%)	1 (1.2%)	1 (3.4%)	0 (0%)	0.994
N/V	15 (11%)	0 (0%)	1 (3.8%)	11 (13.7%)	3 (10.3%)	0 (0%)	0.691
Sweating	29 (21.2%)	0 (0%)	7 (26.9%)	17 (21.2%)	5 (17.2%)	0 (0%)	0.518
Cold Peripheries	12 (8.7%)	0 (0%)	2 (7.6%)	9 (11.2%)	1 (3.4%)	0 (0%)	0.092
Dizziness	9 (6.5%)	0 (0%)	1 (3.8%)	7 (8.7%)	0 (0%)	1 (100%)	0.583
GBW	5 (3.6%)	0 (0%)	2 (7.6%)	1 (1.2%)	1 (3.4%)	1 (100%)	0.024

The study also analyzed the prevalence of symptoms associated with ACS. Table 1 also shows that chest pain was the most common symptom reported (70% of patients), predominantly in the 30-49 years age range (88.4%). Shortness of breath was reported by 37.2% of patients, with higher proportions in the 70-89 years age range (44.8%). While no significant associations were found between other symptoms (palpitations, syncope, nausea and vomiting (N/V), sweating, cold peripheries, dizziness, and generalized body weakness (GBW)) and ACS, the study provides valuable insights into age-related patterns of symptoms and risk factors in ACS patients. Overall, the findings highlight the significance of age and gender in understanding ACS and underscore the importance of chest pain and shortness of breath as predominant symptoms in ACS patients.

Significant associations were found between age groups and certain risk factors as seen in Table 2. Diabetes mellitus (DM) was seen maximum in the age group 50-69 years old (p = 0.041), and hypertension (HTN) also showed a similar pattern with the maximum in the age group 50-69 years (p = 0.006). Another significant association was found between a family history of CAD and the occurrence of ACS, with the highest percentage in the 50-69 years age group (44.5%, p = 0.021). Obesity had the highest prevalence in the 50-69 years age group (p = 0.046). However, no significant associations were found between age and coronary artery disease (CAD), dyslipidemia, or smoking (p > 0.05).

**Table 2:** Risk factors and the significant associations among them. DM = diabetes mellitus; HTN = hypertension; CAD = coronary artery disease.

Risk Factors	Overall	10-29 age group	30-49 age group	50-69 age group	70-89 age group	90-99 age group	P-value
DM	70 (51%)	0 (0%)	7 (10%)	47 (67.2%)	15 (21.4%)	1 (1.4%)	0.041
HTN	99 (72.2%)	0 (0%)	13 (13.2%)	62 (62.6%)	24 (24.2%)	0 (0%)	0.006
Obesity	5 (3.6%)	0 (0%)	2 (40%)	3 (60%)	0 (0%)	0 (0%)	0.046
CAD	71 (51.8%)	0 (0%)	12 (16.9%)	41 (59.2%)	16 (22.5%)	1 (1.4%)	0.654
Dyslipidemia	54 (39.4%)	0 (0%)	1 (1.8%)	39(72.2%)	14 (26%)	0 (0%)	0.927
Smoking	8 (5.8%)	0 (0%)	1 (12.5%)	4 (50%)	2 (25%)	1 (12.5%)	0.810
Family History of CAD	45 (32.8%)	1 (2.3%)	7 (15.6%)	20 (44.5%)	15 (33.4%)	2 (4.4%)	0.021

As seen in Table 3 the analysis also focused on two other variables: raised Trop I (troponin I) and an ECG suggestive of ACS. Among the overall patient population, 94.2% had raised Trop I levels, with varying percentages observed across age groups: 10-29 years (0.7%), 30-49 years (18.6%), 50-69 years (60%), 70-89 years (21%), and 90-99 years (0.7%). However, no statistically significant difference was found in the distribution of raised Trop I across these age groups (p-value = 0.921). 65.6% of the overall patients had electrocardiograms indicating ACS, i.e., they had ST elevation, ST depression, and T wave inversions. Across the age groups, the distribution was as follows: 10-29 years (1.1%), 30-49 years (18.8%), 50-69 years (57.8%), 70-89 years (22.3%), and no patients were available in the 90-99 years age group. The analysis did not reveal a statistically significant difference in the distribution of ECGs suggestive of ACS among the age groups (p-value = 0.628).

**Table 3:** Trop I and ECG findings suggestive of ACS Trop I = troponin I; ACS = acute coronary syndrome; ECG = electrocardiogram.

Variable	Overall	10-29 age group	30-49 age group	50-69 age group	70-89 age group	90-99 age group	P-value
Raised Trop I (n,%)	129 (94.2%)	1 (0.7%)	24 (18.6%)	76 (60%)	27 (21%)	1 (0.7%)	0.921
ECG suggestive of ACS (n,%)	90 (65.6%)	1 (1.1%)	17 (18.8%)	52 (57.8%)	20 (22.3%)	0 (0%)	0.628

## Discussion

This study aimed to examine symptoms and risk factors associated with ACS in different age groups. A total of 137 patients diagnosed with ACS participated in the study. Most patients (58.3%) fell into the age range of 50 to 69 years. The next largest group comprised 29 patients (36.8%) between the ages of 60 and 79, followed by 26 patients (21.6%) aged 30 to 49. These results demonstrate that ACS is more prevalent among individuals in the middle-aged group (50-69 years old). These findings align with previous studies' findings [10-12].

Furthermore, the results revealed that ACS was significantly more prevalent among male patients, accounting for 60% of the cases, while women comprised a smaller percentage (40%) of the individuals diagnosed with ACS. Furthermore, Naito et al. observed a similar distribution of ACS among genders aged 45-65, corroborating our findings [12]. This finding is in good agreement with previous research studies that have identified male sex as a risk factor for acute coronary syndrome [13-16]. 

Among the studied ACS patients, hypertension emerged as the most prevalent risk factor, with 99 cases (72.2%) affected by this condition. In addition, other common risk factors included coronary artery disease (52%), diabetes (51%), dyslipidemia (39.4%), family history (32.8%), smoking (5.8%), and obesity (3.6%). These findings align with previous studies that have identified smoking, hypertension, diabetes, obesity, and dyslipidemia as significant risk factors for the development of ACS [16-18]. These risk factors were highest in the 50-69 age group, ranging from 44-72%.

Among the studied ACS patients, the most prevalent symptom was chest pain, predominantly in the 30-49 age range (88.4%), with minimal presentation in the age group >70. These findings are in harmony with the study mentioned, which stated that while younger adults have higher odds of exhibiting classic ACS symptoms, the elderly have been shown to present with fewer and more ambiguous symptoms, including nausea and dyspnea. [19]

Troponins T and I, both clinically equivalent, demonstrate a sensitivity of 79% to 83% and a specificity of 93% to 95% in detecting myocardial injury [20,21]. While troponin I levels showed varying increases across different age groups, our study did not find a significant association. However, it is noteworthy that in the 90-99 age cohort, patients exhibited normal ECG findings but elevated troponin levels, indicating that troponin may be a superior marker compared to ECG in older individuals [21,22]. A study by Möckel M highlighted that biomarker-based diagnostics are often crucial to deciding whether early imaging is needed or early discharge is possible after the initial clinical evaluation of ACS [23].

While this study provides valuable insights into age-related patterns of symptoms and risk factors in ACS, there are several limitations to consider. Firstly, the study's retrospective design relies on data from patient records, which may contain incomplete or missing information, leading to potential bias. Secondly, the study was conducted at a single medical institute, which may limit the generalizability of the findings to a broader population. Additionally, the sample size of 137 patients may be relatively small, warranting caution in interpreting the results.

Therefore, future directions include conducting a prospective, multicenter study with a larger sample size to enhance generalizability and statistical power. Long-term follow-up of patients could help assess the outcomes and prognostic implications of the identified age-related patterns. Furthermore, investigating the impact of socioeconomic and lifestyle factors on ACS in different age groups could offer further insights into the underlying mechanisms and potential preventive strategies.

## Conclusions

In conclusion, this retrospective, cross-sectional study sheds light on age-related patterns of symptoms and risk factors in patients diagnosed with ACS. The findings highlight the significant associations between age and ACS, as well as gender and ACS. A significant association of p=0.037 was seen between age group 50-69 years and ACS. Another significant association p= 0.019 was seen between the male gender and ACS. Chest pain emerged as the predominant symptom with a significant association of p=0.029 between chest pain and patients of ACS in the age group 50-69 years. while raised troponin I levels were prevalent across all age groups. Moreover, specific risk factors such as diabetes mellitus, hypertension, and a family history of CAD showed a significance of p=0.04, p=0.006, and p=0.021, respectively, with the age group 50-69 years. The study underscores the importance of considering age and gender in ACS management and emphasizes the need for further research to explore the underlying mechanisms and optimize preventive strategies for different age groups. These findings contribute to our understanding of ACS in the context of age-related patterns and inform clinical decision-making and patient care.
